# Cortical Thinning in Patients with Recent Onset Post-Traumatic Stress Disorder after a Single Prolonged Trauma Exposure

**DOI:** 10.1371/journal.pone.0039025

**Published:** 2012-06-13

**Authors:** Yang Liu, Yi-Jun Li, Er-Ping Luo, Hong-Bing Lu, Hong Yin

**Affiliations:** 1 Department of Biomedical Engineering, Fourth Military Medical University, Xi'an, Shaanxi, China; 2 Department of Radiology, Xijing Hospital, Xi'an, Shaanxi, China; Hangzhou Normal University, China

## Abstract

Most of magnetic resonance imaging (MRI) studies about post-traumatic stress disorder (PTSD) focused primarily on measuring of small brain structure volume or regional brain volume changes. There were rare reports investigating cortical thickness alterations in recent onset PTSD. Recent advances in computational analysis made it possible to measure cortical thickness in a fully automatic way, along with voxel-based morphometry (VBM) that enables an exploration of global structural changes throughout the brain by applying statistical parametric mapping (SPM) to high-resolution MRI. In this paper, Laplacian method was utilized to estimate cortical thickness after automatic segmentation of gray matter from MR images under SPM. Then thickness maps were analyzed by SPM8. Comparison between 10 survivors from a mining disaster with recent onset PTSD and 10 survivors without PTSD from the same trauma indicates cortical thinning in the left parietal lobe, right inferior frontal gyrus, and right parahippocampal gyrus. The regional cortical thickness of the right inferior frontal gyrus showed a significant negative correlation with the CAPS score in the patients with PTSD. Our study suggests that shape-related cortical thickness analysis may be more sensitive than volumetric analysis to subtle alteration at early stage of PTSD.

## Introduction

Post-traumatic stress disorder (PTSD) is an anxiety disorder that develops after exposure to a terrifying event or ordeal in which grave physical harm occurred or was threatened. Neuroimaging studies have identified a number of structural and functional alterations associated with PTSD. Structural studies based on magnetic resonance imaging (MRI) have typically indicated structural modifications of brain in several regions of gray matter in patients with PTSD. Most of the studies focused on atrophy of the hippocampus, which is involved in memory processing and regulation of stress [1], [2]. Some other volumetric alteration possibly related to PTSD were also identified, such as amygdala [3], anterior cingulate gyrus [4], insula [5], [6], and cerebellum [7]. Controversially, a series of studies have not found volume deficits in hippocampus [4], [8], amygdala [9], et al, suggesting that PTSD may not induce any structure deficits. Meanwhile, functional neuroimaging studies have also identified altered activities in a series of brain areas, such as less activated in precuneus (located in parietal lobe), which is known to play a role in memory processing [10], while some other studies reported that the parietal lobe was activated and hippocampus was less activated in patients with PTSD [11]. These discrepancies may result from various study characteristics, such as sampling variation (sample size, education, age, and gender), traumatic experiences (combat-related or civilian, single or repeated), disorder type (acute, chronic, or delayed onset), severity of trauma exposure, acquisition time and scanning protocols for MRI (modality, parameter), and different analysis methods [12]. A recent study reported that different types of traumatic experiences may result in different levels of PTSD severity and display distinct PTSD symptom patterns [13]. Moreover, according to the studies mentioned above, the most consistent results on structural atrophy were found in subjects who experienced repeated traumas of short duration, such as combat or abuse-related experience. Few studies have investigated the effect of recent onset PTSD induced by a single prolonged trauma exposure. To reveal the subtle alteration at the early stage of PTSD induced by the prolonged mine disaster, a more sensitive and effective analysis method is needed.

The cerebral cortex is the layer of the brain often referred to as gray matter. Based on the definition of morphological volume, the total volume of gray matter is determined by both subcortical surface and cortical thickness. Though there is a close relationship between the cortical thickness and the volume of gray matter, the difference between them exists in PTSD analysis, when referred to different patterns of physiological and/or pathological changes. For example, based on voxel-based morphometry (VBM) analysis, only shape changes but no volume deficits were identified in recent onset PTSD, suggesting that shape-related thickness analysis exhibits subtle structural changes and may reveal more details than volumetric analysis in the study of recent onset PTSD [14], [15].

At present, alteration of cortical thickness can be used as diagnostic indicators for several brain disorders. For example, Alzheimer's disease is related to pronounced cortical thinning [16], Williams syndrome patients exhibit a significant increment of cortical thickness (5∼10%) in some specific regions [17], and lissencephalic patients present a significant thickening in the frontal lobes [18]. It has also been demonstrated that cortical thickness analysis could be used to investigate subtle structural changes in the brain for the relationship of cognitive abilities and effects of PTSD [19]. Up to now, only two studies have investigated the alteration of cortical thickness related to PTSD. The first one, based on MRI data of veterans, identified the reduction of cortical thickness in bilateral prefrontal cortex and left superior temporal gyrus [19] using a region-of-interest (ROI)-based semi-automatic analysis performed only on the prefrontal cortex. The other study, using the Freesurfer package, found no significant difference in cortical thickness between women with sexual abuse-related PTSD and controls [12]. To our knowledge, no studies have investigated the alteration in cortical thickness related to recent onset PTSD induced by a single prolonged trauma exposure.

The severity of trauma exposure is thought to be the key factor for assessing the risk for adverse outcomes [15], [20]. Some studies have paid attention to the relationship between PTSD severity and structural alteration, and found that the severity of PTSD symptoms is associated with smaller volumes of hippocampus [21], [22] and left amygdala [3] in patients with PTSD. In addition, the PTSD severity was found to be negatively correlated with gray matter density in limbic structures [23]. All these results support that PTSD severity is associated with structural changes in the limbic system. However, up to now, no analysis has been reported to investigate the relationship between cortical thickness and PTSD severity.

Cortical thickness in MRI data can be automatically estimated by either surface-based methods, voxel-based methods, or a mixture of the two. Recently, Hutton *et al.* proposed a method for automatic voxel-based cortical thickness estimation based on the Laplacian method [24], which has been validated by repeated MRI scans and simulated data, and is regarded as a precise and reliable tool for thickness analysis of *in vivo* brain MRI data [25]. The method could be used with statistical parametric mapping (SPM) to detect small differences in cortical thickness between two groups of subjects [24]. However, due to the complicated numerical solution used for the Laplace's equation, the procedure would be time consuming and sometimes encounter difficulty converging to a stable solution. If the improved Laplacian method presented in [25], [26] could be adopted in the procedure, which integrates a fast numerical solution with partial differential equation (PDE) for accurate estimation [26], [27], the thickness estimation would be more effective and robust. In addition, the DARTEL normalization provided by the newer version of VBM package can register inter-subject of brain images more precisely [28], exhibiting more accurate realignment of subtle structures [29] and a higher sensitivity in detecting thin elongated structures like hippocampal abnormalities [30]. The inclusion of VBM-DARTEL for spatial normalization in the procedure may further improve the analysis on subtle alteration of structures, such as that on cortical thickness.

The present study aimed to explore the differences in cortical thickness between survivors with recent onset PTSD and survivors without PTSD from the same coal mine flood disaster, and the relationship between cortical thickness of identified regions and the PTSD severity. To measure the complex three-dimensional (3D) cortical thickness accurately, the improved Laplacian method was applied on gray matter of subjects segmented by VBM-DARTEL. Then the thickness maps were analyzed by SPM to find structural alterations in patients with recent onset PTSD, compared to a non-PTSD group. Finally, ROI analysis was used to investigate the relationship between cortical thickness of identified regions and the PTSD symptom severity in the patients with PTSD.

## Methods

### Subjects

The subjects in this study completely overlap with those in the previous volumetric study of recent onset PTSD [15]. Briefly, a total of twenty right-handed male survivors from the same coal mining flood disaster which occurred in 2007 in the Henan province of China were included in this study. In the mining disaster, 69 miners were trapped for 72 hours, and all of them were rescued and survived. Forty-eight out of 69 survivors were hospitalized and received a medical checkup. Six months later, 17 out of the 48 survivors met the diagnostic criteria for PTSD, and among them, 10 agreed to participate in this MRI study. All ten PTSD patients were diagnosed with DSM-IV [31] and the Structured Clinical Interview for DSM-IV (SCID) [32]. The severity of their symptoms was assessed with the Chinese version of the Clinician-Administered PTSD Scale (CAPS) [33]. In addition, 10 out of 31 survivors without PTSD agreed to participate in the MRI study as a non-PTSD group. There were no significant differences in symptom severity, age and education level between these subjects and others who did not join the study. The elapsed time between the traumatic event and MRI scans ranged from 187 to 190 days.

With the recruited PTSD and non-PTSD groups in this study, each group had 10 male subjects. The identified PTSD subjects had never received any psychiatric treatment before. Moreover, none of the subjects had a history of treatment with psychotropic drugs or of substance (alcohol, smoking or drug) abuse. The study was approved by the Institutional Board of The Fourth Military Medical University. All subjects received a comprehensive description of the MRI study and gave voluntarily written informed consent before entering the study.

### MRI acquisition

MRI examinations were conducted at the Radiology Department of Xijing Hospital. All the scans were acquired on a 3.0 T MR scanner (MAGNETOM Trio, Siemens AG, Erlangen, Germany). A high-resolution 3D magnetization prepared rapid acquisition gradient echo (MPRAGE) T1-weighted sequence covering the whole-brain (176 sagittal slices) was acquired. Other acquisition parameters were set as TR = 1900 ms, TE = 2.26 ms, TI = 900 ms, flip angle = 9°, acquisition matrix = 256×256, field of view = 220 mm, and 1.00 mm slice thickness with no inter-slice gap.

### Data analysis

VBM image analysis was performed on a computer workstation installed with MATLAB 7.11.0 and VBM8 package (http://dbm.neuro.uni-jena.de/vbm) under SPM8 (Wellcome Department of Imaging Neuroscience, London, UK; http://www.fil.ion.ucl.ac.uk/spm/software/spm8/).

In this study, the individual MRI scan was first spatially normalized to a template space with high-dimensional DARTEL normalization, which is suitable for the analysis of subtle shape alteration such as cortical thickness, compared to the standard normalization in SPM. Normalized images were then segmented into gray matter, white matter and cerebrospinal fluid (CSF) with signal intensity and prior probability information.

### Thickness estimation

**Figure 1:**
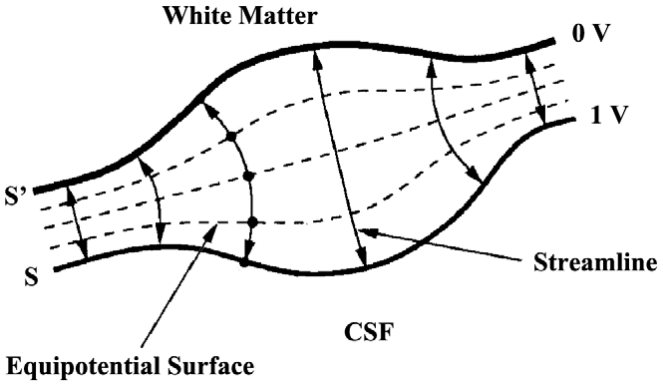
Two-dimensional example of Laplacian method. The Laplace's equation is solved between S and S′, which have predetermined boundary conditions of 1 V and 0 V, respectively (as in an electric field). The length of the streamlines between the two surfaces then defines the thickness.

Based on the segmented gray matter, white matter and CSF, the 3D cortical thickness was estimated by the Laplacian approach. The Laplace's equation, a second order PDE, was used to define the thickness of the cortex and can be expressed by
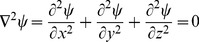
(1)where *ψ* is the scalar field [34]. In the present study, the potential of white matter voxels was set to 0 V and that of CSF voxels was set to 1 V, respectively. Therefore, the potential value of *ψ* on the boundary surface *S* was 1 and that on the boundary surface *S′* was 0, as shown in Figure 1.

The Laplace's equation was then solved in the volume of gray matter by a newly-proposed numerical solution for 3D MRI, which is accurate and computationally fast [26]. This method is based on the representation of the 3D potential function as a simple linear correlation of three 2D potential functions. Once the solution of *ψ* is obtained, the gradient of scalar field at each point can then be normalized to produce a unit tangent field 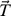, as shown below:
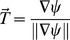
(2)where 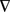 represents the gradient operator.

After the computation of 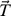, the cortical thickness or the streamline could be computed for each voxel inside the cortical volume. In this step, the iterative solution of a pair of first-order linear PDEs was used for an accurate, fast and stable estimation, based on the work of Yezzi *et al*. [27].

### Sulci detection and thickness correction

The cerebral cortex is a topological shell of gray matter surrounding a core of white matter with less than half of the cortical surface visible as gyri and the majority is buried in sulci [35]. However, due to noise, intensity inhomogeneities, and partial volume effect in MRI images, it is hard to classify the tissues perfectly, especially in sulci regions. Considering the importance of accurate segmentation of gray matter on thickness estimation, mixture-based segmentation with probability maps was performed to get accurate detection of the cortex, especially for regions in deep sulci. In this study, the algorithm developed for sulci detection and thickness correction was employed to further improve the brain segmentation after the thickness estimation [36]. Sulci detection involved region growing in areas of which thickness was higher than what is expected for the cortex (the cortical thickness is known less than 5 mm) [36], [37]. Information from the unit tangent field in Eq. (2) was also used to control the directions of region growing and the output probability maps of the segmentation process was used to decide which voxel belongs to white matter, gray matter, or CSF. After the correction of the segmentation, the cortical thickness was re-calculated accordingly and more accurate result was obtained for the whole brain.

### Statistical analysis

Group differences in demographic variables were examined with independent *t*-tests by SPSS 13.0 (SPSS Inc., Chicago, IL).

Typical values for cortical thickness in adult humans are between 3 and 5 mm and a decrease of 16 µm per decade could be observed during aging [38]. Thus, group comparisons of cortical thickness were performed using analysis of two sample *t*-test in SPM8 with age as covariates to account for the age effect. Before statistical analysis, all cortical thickness maps were smoothed by an isotropic Gaussian kernel with the full width at half maximum of 8 mm. Two planned contrasts were examined as follows: PTSD>non-PTSD subjects and PTSD< non-PTSD subjects. The statistical significance for differences in thickness tested at the voxel level was set to an uncorrected *p* value of 0.001.

After statistical analysis of cortical thickness between the PTSD group and the non-PTSD group, ROI analysis was performed to investigate the relationship between the identified regions and PTSD symptom severity in patients with PTSD. According to the definition of cortical thickness in this study, the cortical thickness is arc length of the streamline from the inner boundary to the outer boundary, which means that every voxel on the streamline across the identified region may have an effect on the statistical analysis. To avoid possible bias induced by these voxels on the streamline, the region that consists of all the voxels on streamlines across the identified region was used for ROI analysis. To get this region, the information from the unit tangent field in Eq. (2) was used to control the directions of region growing to find all the voxels on these streamlines, and then the masks were generated. After the generation of the mask, dot product of the cortical thickness map and the masks was performed to generate the ROIs in MATLAB. Then the mean thickness of the generated ROI was calculated.

ROI-based correlational analysis between mean cortical thickness of generated ROIs and PTSD symptom severity was performed in the patients with PTSD using Pearson's partial correlation analysis in SPSS 13.0. Age was treated as a controlling covariant and the significance level was set at *p<*0.05.

Using Talairach Client software developed by Lancaster *et al.*, the peak MNI coordinates was transformed to the Talairach space for region identification [39], [40].

## Results

### Subject demographics

Table 1 gives the mean and standard deviation of physical and clinical characteristics for all subjects. All subjects in this study came from the same community and did not differ significantly in socioeconomic status. The two groups (PTSD and non-PTSD) significantly differed in age. As expected, the PTSD group had higher scores on the CAPS than non-PTSD group. And there was no significant difference in the level of education.

**Table 1:** Subject physical and clinical characteristics.

	PTSD	non-PTSD	t tests	
			*t value*	*P*
Age (years)	40.80±6.83	34.30±5.37	2.318	0.032
Education level	6.67±2.06	7.25±2.38	1.72	0.14
CAPS^a^	78.72±17.2	31.40±18.57	6.306	<0.001

a CAPS: Clinician-Administered PTSD Scale, with higher scores indicating greater PTSD severity (Wu and Chan, 2004).

### Cortical thickness

The mean cortical thickness and the percentage of cortical voxels with thickness greater than 5 mm in PTSD and non-PTSD groups, before and after the sulci detection and thickness correction, are displayed in Table 2. The results indicate that the mean cortical thickness of subjects with PTSD was almost the same as that of non-PTSD. Thickness correction based on sulci detection reduced the number of voxels with erroneously higher thickness (>5 mm) greatly by a decrease over 40%.

**Table 2:** Thickness results with and without sulci detection.

	PTSD		non-PTSD	
	Thickness (mm)	Thickness>5 mm (%)	Thickness (mm)	Thickness>5 mm (%)
no sulci detection	5.58±0.28	45.8±5.79	5.69±0.35	49.6±5.81
sulci detection	4.16±0.13	4.82±0.51	4.19±0.16	5.15±0.69

### Cortical thickness analysis

**Figure 2:**
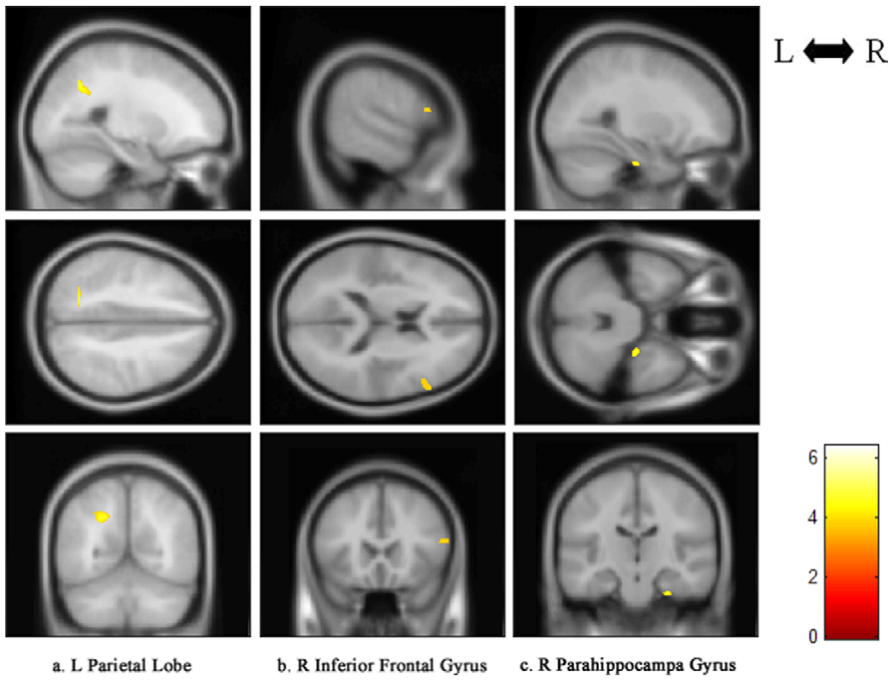
Regional cortical thinning in recent onset PTSD patients versus trauma survivors without PTSD. Regional cortical thinning in the following areas were rendered onto orthogonal slices: (a) left parietal lobe; (b) right inferior frontal gyrus; (c) right parahippocampal gyrus. L, left hemisphere; R, right hemisphere. PTSD, post-traumatic stress disorder.

Comparisons of cortical thickness in survivors experienced mining disaster with and without PTSD are shown in Table 3 and Figure 2. It indicated that in the left parietal lobe, right inferior frontal gyrus, and right parahippocampal gyrus, cortical thickness of subjects with PTSD was obviously thinner than those without PTSD.

**Table 3:** Significantly thinning cortical regions between PTSD and non-PTSD groups.

Brain regions	MNI^a^ (x,y,z)	BA^b^	Cluster size	t score
Left parietal lobe (precuneus)	−22, −61, 39	7	748 (376)	4.85
Right inferior frontal gyrus	57, 22, 16	45	224	3.84
Right parahippocampal gyrus	27, −18, −32	36	124	4.85

a MNI: Montreal Neurological Institute system.

b BA: Brodmann Area.

Based on brain atlas, the region of precuneus is located in the parietal lobe. With the transform provided by Talairach client, the peak MNI coordinates (−22, −61, 39) is located inside the precuneus. Thus, precuneus is also identified as significantly thinner region.

### Influence of symptom severity

**Figure 3:**
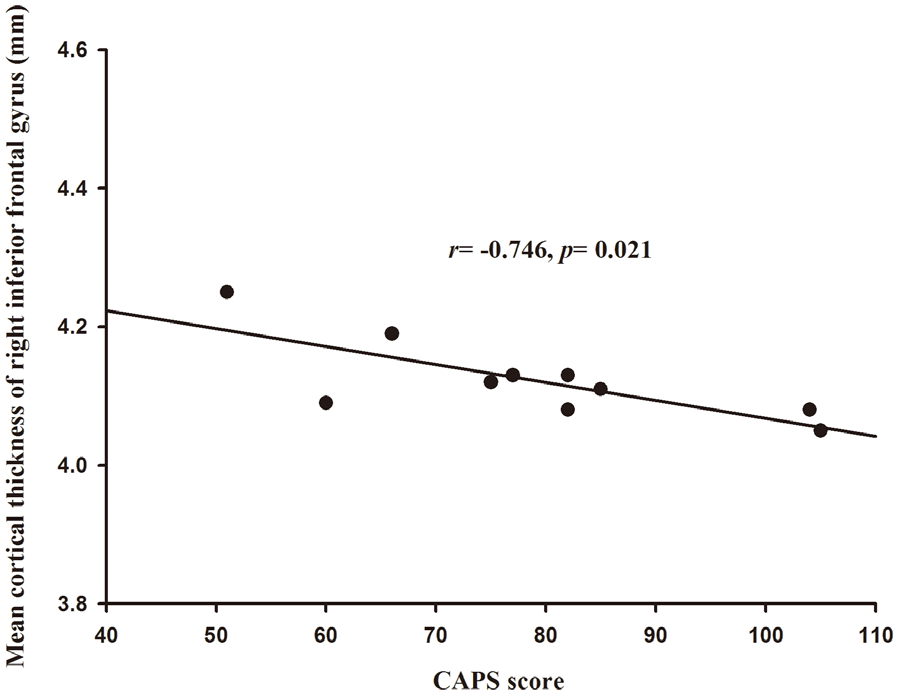
Mean cortical thickness of the right inferior frontal gyrus correlations with the CAPS score of PTSD patients. PTSD, post-traumatic stress disorder. *r*, Pearson's correlation coefficient; *p*, *p* value.

The mean cortical thickness derived from ROI analysis tended to correlate negatively with the CAPS score in the right inferior frontal gyrus (*r* = −0.746, *p* = 0.021), as shown in Figures 3, while no significant correlation was found in the left parietal lobe and right parahippocampal gyrus. This result indicates that the cortical thinning in the right inferior frontal gyrus was associated with the symptom severity of the disorder in subjects exposed to severe trauma.

## Discussion

The present study analyzed the difference in cortical thickness of whole brain in patients with recent onset PTSD due to a mining disaster. To perform the analysis, a thickness estimation procedure that combined SPM8, Laplacian method and sulci detection was presented to segment 3D cortex from MRI images, and estimate and analyze cortical thickness maps throughout the whole brain more accurately. After the comparison of cortical thickness between subjects with and without PTSD, the relationship between cortical thickness of identified regions and the PTSD symptom severity was investigated for the first time.

In neuroanatomical studies, cortical thickness is a morphological index to describe the combined thickness of the layers of the cerebral cortex, which correlates with the size, density, and arrangement of neurons, glial cell and nerve fibers [19]. Moreover, it is associated with cognitive abilities [41]. The cortical thinning might be a reflection of structural alteration in the boundary between gray matter and white matter, which is related to change of myelination in specific regions of brain [42].

In this study, we found that recent onset PTSD was associated with cortical thinning in the left parietal lobe, right inferior frontal gyrus, and right parahippocampal gyrus, compared with matched subjects without PTSD. In addition, regional cortical thickness of right inferior frontal gyrus showed a significant negative correlation with the CAPS score in the patients with PTSD. However, in our previous volumetric study, gray matter volume deficits in the left anterior hippocampus, left parahippocampal gyrus, and bilateral calcarine cortex were identified [15]. The discrepancy may come from different analysis methods used in these two studies. The high-dimensional DARTEL normalization in VBM8 was used for accurate realignment and sensitive detection of subtle structures in this study. More importantly, it may come from different properties of volumetric and cortical thickness analysis. The volumetric analysis focuses more on the alteration from a global aspect, while the thickness one pays more attention to subtle and detailed changes.

Our finding of cortical thinning in the left parietal lobe, right inferior frontal gyrus, and right parahippocampal gyrus is in line with several neuroimaging studies. The parietal lobes are thought to be involved in temporal and spatial orientation function [43], which may participate in processing of spatial and temporal information related to a traumatic event [44]. The observation of diminished activation in the parietal lobes during traumatic memory retrieval may provide an explanation to why traumatic memories are experienced as being ‘present tense’ [44]. It has been also reported that precuneus (located in the parietal lobe) is associated with source memory processing [45]. Especially, the left precuneus is related to mental imagery and posterior buffer working memory [10], [46]. Based on functional neuroimaging studies, diminished activity in left parietal lobe [47] and precuneus [48] was identified in patients with chronic PTSD. However, to our knowledge, no structural studies have reported structural alteration of parietal lobe and/or precuneus so far. The finding of a thinner left parietal lobe in this study may identify a potential region with structural alteration related to recent onset PTSD.

It is common that patients with PTSD have difficulties in verbally describing and processing trauma-related experiences [1]. The anterior language area is a part of the inferior frontal gyrus. Deficit in the inferior frontal gyrus was noticed in patients with chronic PTSD [49], and thinner cortical thickness of the inferior frontal gyrus was also identified in veterans with PTSD [19]. The inferior frontal gyrus has attracted special attention in functional neuroimaging studies. It was reported that an increased [50], [51] or a decreased [52] activation in right inferior frontal gyrus was identified in patients with chronic PTSD, while decreased activation in right inferior frontal gyrus was identified in patients with acute PTSD [1]. In this study, thinner right inferior frontal gyrus was observed in patients with resent onset PTSD, indicating a possible relation of this region with acute or recent onset PTSD. Further research with larger sample size and various disorder types is needed for better explanation of the observed inconsistence in this region.

Previous studies on structural alteration of the brain in patients with PTSD have identified atrophic hippocampus, which is critically associated with explicit (declarative) memory, working memory [53], memory for episodic events [54], and regulation of stress [1], [2]. The parahippocampal gyrus is a cortical region that surrounds the hippocampus, which may play an important role in episodic, spatial, and contextual memory, as well as the encoding and recognition of emotional scenes [15], [55], [56]. Deficit in parahippocampal gyrus was identified in patients with chronic PTSD [49]. Studies based on fMRI observed lower activations in the right parahippocampal gyrus in acute PTSD patients when performing memory recall tasks compared with symptom provocation tasks, indicating that the ability of active memory recall, a kind of active working memory, was impaired in subjects with acute PTSD induced by the coal mine disaster [1].

The ROI analysis performed in this study indicates that regional cortical thickness of right inferior frontal gyrus has a significant negative correlation with the CAPS in the patients with PTSD. The severity of the PTSD symptoms was found negatively correlated with gray matter volume of the left amygdala [3], left hippocampus [57] and bilateral hippocampus in chronic PTSD [22], [58]. But no study on the relationship between the cortical thickness and PTSD severity has been reported previously. According to the definition by the Laplacian method, the cortical thickness is the arc length of the streamline from the inner boundary to the outer boundary, which means that every voxel on streamlines that across the significantly thinner region may have an effect on the statistical analysis. Therefore, in this study, the region that consists of all the voxels on streamlines across the significantly thinner region was used in this study to investigate the relationship between cortical thickness and PTSD symptom severity, though doing so might make the correlation analysis less significant than that using only the region with significant difference. That may be one reason for insignificant correlation between CAPS and regional cortical thickness of left parietal lobe and right parahippocampal gyrus after the correlation analysis.

For subjects surviving the coal mining flood disaster, we could not control the sample size, just like some studies on PTSD induced by other sudden disasters, such as a fire disaster [5] and sarin attacks [3], [59]. However, we've tried our best to make all data consistent to alleviate possible sampling bias. Meanwhile, it has been noticed that quite a few studies related to sudden disasters could not survive after multiple comparisons [3], [5], [59]. As the false positive rate is controlled more conservatively for multiple comparison correction, statistical power may decrease [60]. It is true that uncorrected results have relatively higher false positive rates compared with corrected results, but it can be interpreted as possible differences and/or trends in nature [61]. In this study, the results could not survive after multiple comparison correction. That may be due to the small sample size used in this study, and subtle alteration of cortical thickness caused by early stage of PTSD. Considering the relatively low *p* value of 0.001 used here, we believe that the difference in cortical thickness between two groups is statistically reasonable.

It has been widely reported that mean cortical thickness decreases with aging [38], [62]. That's why the Pearson's partial correlation analysis was used in this study to reflect the relationship between CAPS score and mean thickness of identified regions with age effect removed. However, the extent of cortical thinning seems quite different in different regions [62]. For example, significant effect of age on inferior frontal gyrus has been reported frequently [62], [63], while age effect on cortical thickness of the parietal lobe and parahippocampal gyrus was rarely observed [63]. For the patients with PTSD used in this study, no significant correlation between mean cortical thickness of generated ROIs and age was observed using Pearson's correlation analysis (*p<*0.05), which may be due to the narrow age span from 35 to 45 of most PTSD patients. To demonstrate the direct relationship between CAPS score and mean cortical thickness, ROI analysis by Pearson's correlation was also performed with age effect ignored. It demonstrated that the mean cortical thickness tended to correlate negatively with the CAPS score in the left parietal lobe (*r* = −0.673, *p* = 0.033), right inferior frontal gyrus (*r* = −0.762, *p* = 0.010), and right parahippocampal gyrus (*r* = −0.661, *p* = 0.037). Stronger correlation between the symptom severity and the mean thickness of identified regions could be observed when age effect was ignored.

Several limitation of this study should be addressed. First, the sample size is small. Second, all the participants were trauma-exposed and no normal subjects without PTSD were included. Further study is underway to investigate the effects of trauma exposure by adding a non-exposed control group.

In conclusion, the present study identifies the structural changes of cortical thickness in survivors with and without PTSD. The results of cortical thinning in regions related to memory processing and regulations of stress indicate that shape-related thickness analysis may be more sensitive than volumetric analysis to subtle alteration at early stage of PTSD. The negative correlation between the CAPS and cortical thickness of the right inferior frontal gyrus indicates that the cortical thinning in this region may be associated with the symptom severity of stress disorder. In addition, the proposed pipeline for cortical thickness analysis that integrates VBM-DARTEL, SPM and improved Laplacian method offers a useful and automated way for neuroanatomical studies. Prospective studies should be carried out in larger sample sizes with well-matched subjects to achieve more completely reliable findings.
